# Neural Correlates of the Difference between Working Memory Speed and Simple Sensorimotor Speed: An fMRI Study

**DOI:** 10.1371/journal.pone.0030579

**Published:** 2012-01-23

**Authors:** Hikaru Takeuchi, Motoaki Sugiura, Yuko Sassa, Atsushi Sekiguchi, Yukihito Yomogida, Yasuyuki Taki, Ryuta Kawashima

**Affiliations:** 1 Smart Ageing International Research Center, Institute of Development, Aging and Cancer, Tohoku University, Sendai, Japan; 2 Department of Functional Brain Imaging, Institute of Development, Aging and Cancer, Tohoku University, Sendai, Japan; 3 Division of Developmental Cognitive Neuroscience, Institute of Development, Aging and Cancer, Tohoku University, Sendai, Japan; 4 Japan Society for the Promotion of Science, Tokyo, Japan; 5 Brain Science Institute, Tamagawa University, Tokyo, Japan; Cuban Neuroscience Center, Cuba

## Abstract

The difference between the speed of simple cognitive processes and the speed of complex cognitive processes has various psychological correlates. However, the neural correlates of this difference have not yet been investigated. In this study, we focused on working memory (WM) for typical complex cognitive processes. Functional magnetic resonance imaging data were acquired during the performance of an N-back task, which is a measure of WM for typical complex cognitive processes. In our N-back task, task speed and memory load were varied to identify the neural correlates responsible for the difference between the speed of simple cognitive processes (estimated from the 0-back task) and the speed of WM. Our findings showed that this difference was characterized by the increased activation in the right dorsolateral prefrontal cortex (DLPFC) and the increased functional interaction between the right DLPFC and right superior parietal lobe. Furthermore, the local gray matter volume of the right DLPFC was correlated with participants' accuracy during fast WM tasks, which in turn correlated with a psychometric measure of participants' intelligence. Our findings indicate that the right DLPFC and its related network are responsible for the execution of the fast cognitive processes involved in WM. Identified neural bases may underlie the psychometric differences between the speed with which subjects perform simple cognitive tasks and the speed with which subjects perform more complex cognitive tasks, and explain the previous traditional psychological findings.

## Introduction

Studies of individual information processing speed (simple processing speed) are traditional and prominent research fields in psychology. Processing speed has traditionally been measured by how fast individuals execute cognitive tasks, particularly elementary cognitive tasks.

However, psychological characteristics of processing speed measured by simple cognitive tasks and those measured by complex cognitive tasks differ [1] (further details of these differences are explained below). In this study, we aimed to investigate the neural correlates of differences between the processing speed of simple and complex cognitive processes. This study investigates the difference between the processing speed of simple and complex cognitive processes, which is important for three reasons. First, previous psychological studies have shown that the degree of correlation between individual processing speed and psychometric measures of intelligence is positively associated with the level of complexity of the processing speed tasks involved [2], [3], [4], [5]. Second, previous psychological studies on the age-related decline of cognitive abilities suggest a distinction between sensorimotor and cognitive speeds [4], [6], [7], [8]. Cognitive speed, rather than sensorimotor speed, is an important proximal mediator of the adult age-related variance in several higher order cognitive tasks [1]. Furthermore, increases in the complexity of an accelerated cognitive task affect the performance of older adults to a greater degree than that of young adults [1]. Third, the distinction between the speed of complex cognitive processes requiring inhibitory cognitive processes and simple cognitive processes has been well stressed in studies on the circadian rhythms' effect on cognitive function. Psychological studies on the effect of circadian rhythms revealed that an individual or group performance of tasks designed to evaluate complex cognitive speed, but not of tasks to evaluate simple speed, was impaired during a non-optimal time of the day [9]. For example, performance on tasks using the interference card of the Stroop task, in which subjects have to resolve interference, was affected by circadian rhythm, whereas performance on tasks using simple color and word cards did not differ throughout the day [9].

**Figure 1:**
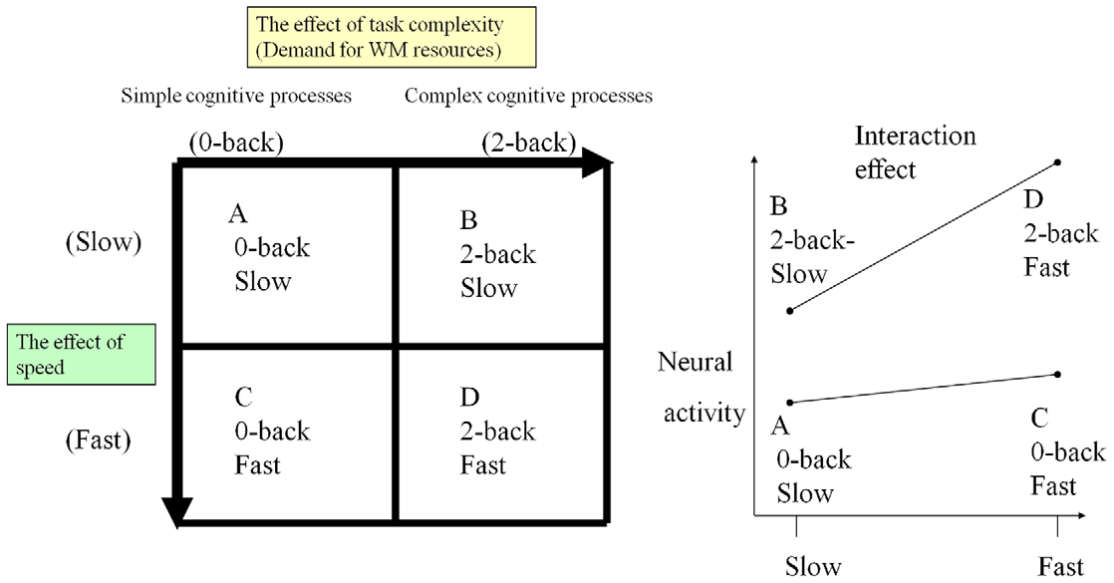
Our conceptual schema of the difference between the effect of speed on simple cognitive processes and on complex cognitive processes, as well as our two factorial task design. We assumed the critical difference between simple and complex cognitive processes (one of the main effects: {(B+D)−(C+A)} was the demand for WM resources, based on previous studies. (C−A) is the effect of speed on simple cognitive processes, and (D−B) is the effect of speed on complex cognitive processes (See the left figure). The purpose of this study was to reveal the neural correlates responsible for the difference in processing speed between simple and complex cognitive processes ({(D−B)−(C−A)} interaction effect in the design. See the right figure).

Processing speed has gathered much attention in psychology because of its correlation with higher order cognitive abilities such as working memory (WM) capacity and psychometric measures of intelligence [10]. Neuroimaging studies have addressed cortical activation, which corresponds to the effect of speed in various cognitive tasks [11], [12], [13], as well as the neural or structural basis of simple processing speed [14], [15], [16] and that of WM [17]. However, differences in the effects of the speed of complex cognitive processes and the speed of simple cognitive processes remain unstudied. Considering the importance of cognitive speed and differences in the effects of the speed of complex cognitive processes and the speed of simple cognitive processes on human psychometric intelligence, aging, and the circadian rhythm, it is important to investigate this issue. The objectives of this MRI study were twofold: to investigate the neural correlates of the difference between simple sensorimotor speed and complex cognitive speed with WM (*i.e.*, the WM-specific speed effect) using functional activity and a functional connectivity analysis, and to reveal the neural basis of an individual's ability to execute fast cognitive processes in WM using morphometry. We used WM tasks for complex cognitive tasks in this study. Complex cognitive processes are considered to be cognitive processes that place a demand on WM resources (See Fig. 1 for our conceptual schema regarding this). This is because, a previous study [1] suggested that demand for WM resources plays a key role in the psychological effect of cognitive complexity.

We hypothesized that the lateral prefrontal cortex (LPFC) would be the neural correlate responsible for the difference between the speed of simple and complex cognitive processes. Our hypothesis was based on previous studies of the psychological correlates of this difference, such as intelligence and cognitive aging. Previous studies of the psychometric measures of intelligence have utilized diverse neuroimaging techniques to demonstrate that intelligence is associated with functional activation in the LPFC [18], the functional connectivity between the LPFC and other regions (primarily the frontoparietal regions at rest) [19], and regional gray matter volume (GMV) in the LPFC [20]. Furthermore, previous functional and structural imaging studies of cognitive aging suggest specific vulnerability in the PFC [21]. While the LPFC is important for intelligence, recent studies indicate the manifestation of intelligence appears to involve the fronto-parietal network of brain regions [22]. Thus, we also predict that the network involving the fronto-parietal regions is the neural correlate responsible for the difference between the speed of simple and complex cognitive processes.

## Methods

### Ethics statement

In accordance with the Declaration of Helsinki (1991), written informed consent was obtained from each subject. This study was approved by the Ethics Committee of Tohoku University.

### Participants

Twenty-three healthy, right-handed males participated in the MRI study. The participants were limited to men to reduce heterogeneity in the data. A discussion regarding heterogeneity in regional structures between sexes can be seen in a previous study [23]. The mean age of the subjects was 21 years (age range 19–28). Data from one subject were excluded from the fMRI and functional connectivity analysis due to excessive motion (>3 mm) during the fMRI task. The mean age of the 22 subjects remaining for the functional MRI and functional connectivity analysis was 21 years (age range 19–24). Handedness was evaluated using the Edinburgh Handedness Inventory [24]. All subjects had normal vision, and none had a history of neurological or psychiatric illness. Intelligence of the sample in the MRI study was not measured.

### Cognitive tasks

**Figure 2:**
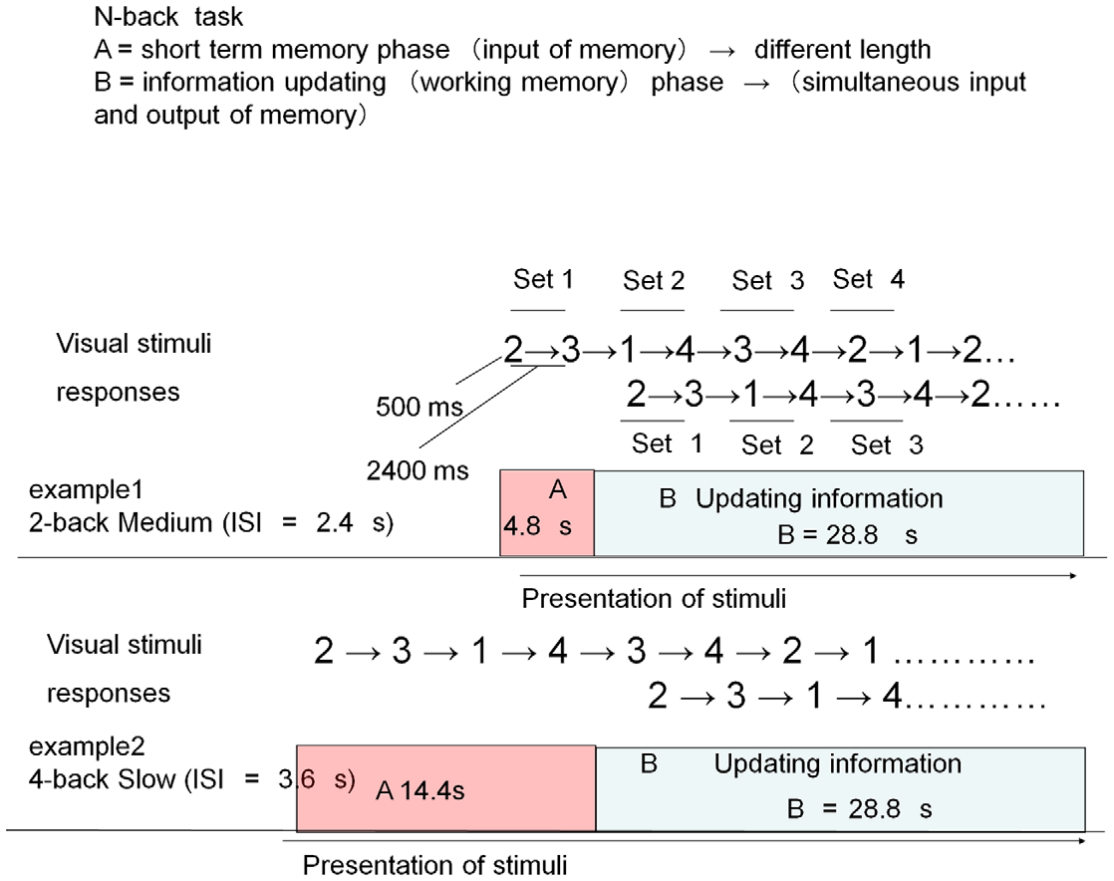
An example of trials illustrating the schematic representation of tasks of different memory loads and the speeds and the strategies used to complete them. Participants were instructed to update memory N by N, not one by one. Each block of the N-back task consisted of phase A and phase B. Phase A was a short-term memory phase in which participants had to memorize presented numbers (input of memory). The length of this phase A differed and depended on the other conditions. Phase B was an information-updating (working memory) phase, and participants had to perform memory input and output simultaneously.

We conducted an MRI study to examine individual responses during the performance of an N-back task, a typical WM task for fMRI studies, in which both the interstimulus interval (task speed) and memory load were varied in an incremental fashion (Fast, Medium, or Slow×0-, 2-, 3-, or 4-back conditions). Participants performed an N-back task using 4 numbers (*i.e.*, 1–4). In the task, the participants were asked to memorize a series of numbers and their temporal order, update the list of recent items, and select the responses that corresponded to the previously observed stimuli, according to the N-back rule (See Fig. 2).

The stimuli consisted of the numbers 1 to 4 shown in a random sequence on the screen (see Fig. 2). Participants had to make responses either directly following the stimulus (0-back), or after a delay of two (2-back), three (3-back) or four (4-back) stimuli. The task level of the memory load, but not the task speed, was shown above the stimuli before the task started for 2.8 s and remained visible during the task period. A single–condition block consisted of a 2.8-s presentation of the task instructions (*e.g.*, “4-back”), followed by various lengths of short-term memory phases, during which subjects had to remember the number presented, and then a 28.8-s updating memory phase, in which participants had to perform the simultaneous input and output of memory (See Fig. 2). The first N trials happened to be short-term memory phases. As short-term memory is thought to differ from WM [25], which involves the manipulation of maintained information, we designed a block according to the rules described above to control the length of the WM phase of the task. To clarify, maintenance has been defined as transferring, maintaining (including rehearsal), and matching information in WM [26], whereas manipulation refers to the additional reorganization or updating of each memory set. Only brain activities from the 28.8-s updating memory phase, in which participants had to perform the simultaneous input and output of memory, were handled in the imaging data analysis. The length of a short-term memory period varied based on the conditions of memory load and task speed {*e.g.*, in the 4-back, Fast (ISI = 1.2 s) condition, the length was 4.8 s; in all 0-back conditions, the length was 0 s}. Each N-back condition was separated by a 16.8-s rest including a 2.8-s queue for the next condition. During the task, the participants were instructed to fix their gaze on the screen unless they had to reconfirm the task level of the memory load. As there are various strategies for performing N-back tasks, the individuals were requested to follow a specific, indicated strategy. Participants were told to update memory N by N (*e.g.*, in the 3-back task, they were asked to update their memory three by three), not one by one. To clarify, in the 3-back tasks, they first had to remember three numbers presented and then, while they pushed the corresponding three buttons, they were asked to remember the three incoming numbers (see Fig. 2).

There were four different conditions for memory load (*i.e.*, 0-, 2-,3-, and 4-back), and three different task speed conditions {Fast (ISI = 1.2 s), Medium (ISI = 2.4 s), and Slow (ISI = 3.6 s)}, for a total of 12 combined conditions overall.

Visual stimuli were presented using Presentation Software (Neurobehavioral Systems, Inc., Albany, CA, USA). Stimuli were projected to participants via a screen positioned at the head-end of the board. Participants viewed the screen through a mirror attached to the head coil. A fiber-optic, light-sensitive key press button box was used to record participants' behavior. It turned out that, for equipment-related reasons, the responses from the fourth button could not always be obtained. Therefore, we analyzed the stimuli of corresponding responses from the remaining three buttons (in other words, the trials where the correct responses require the fourth button were not included in the calculation of performance). This procedure did not change the nature of the behavioral data and chance performance remains at 25 percent. Therefore, we did not change the button-press method of the task.

To ensure that the participants would not anticipate the end of the task, individuals were not informed that the length of the overall blocks (short-term memory and WM phases) was varied, nor were they given any indication of how much time they had to continue the task on each block.

In contrast to other variants of the N-back task, only accuracy was used as a measure of performance in this task. Reaction time did not reflect performance because, in all levels except the 0-back level, participants knew that the next response would be presented before they were allowed to respond, as was determined by the preceding stimuli. For the same reason, we instructed the individuals to emphasize accuracy rather than speed, as has been described elsewhere [27].

### Experimental paradigm

Before the fMRI scanning sessions, subjects were given instructions on the task to be performed. After the instructions, the individuals were allowed a 16-min practice run. The scanning phase of the experiment was divided into three sessions of 21 min each. Each session was composed of two blocks of each condition. In each session, conditions of the different memory loads and conditions of the different task speeds were presented in a fixed order, which made the first half and last half of the session the same. The order of conditions was balanced across all participants. Immediately following the last scanning session, the individuals completed a questionnaire to ascertain the strategies used while performing the task, the subjective difficulty, and their awareness of task speed manipulation. A 16-m practice session was set to learn the task rules and the strategy (as is described in the Cognitive tasks section) well. The 16-m practice was considered necessary because, not only is the N-back task a complex task generally, but also, we controlled the type of strategy utilized by teaching the subjects a specific strategy in this study. Significant noise was detected in the scanned images in a session for a subject, and therefore, another session (fourth scanning session) was conducted for the same subject. We discarded the imaging data from the session in which noise was detected because the data could not be analyzed as a result of the error.

### Behavioral data analysis

The behavioral data were analyzed using the statistic software, SPSS 16.0 (SPSS Inc., Chicago, IL). Behavioral data in the MRI study were analyzed to confirm subject compliance with task performance and the effectiveness of the manipulation of memory load and task speed. The participants' performance was evaluated using 4- (memory load)×3- (task speed) repeated measure ANOVAs, and accuracy measures. We used the Bonferroni adjustment for multiple comparisons in the *post hoc* analysis.

### Image acquisition

Thirty-three transaxial gradient-echo images (echo time = 50 ms, flip angle = 90°, slice thickness = 3 mm, slice gap = 0.99 mm, FOV = 192 mm, matrix = 64×64) covering the entire brain were acquired at a repetition time of 3 s, using an echo planar sequence and a 1.5-T Siemens Symphony MR scanner (Siemens, Erlangen, Germany). Excluding the four dummy scans for stabilization of the T1-saturation effect, 413 volumes were acquired in each of the three fMRI sessions. Three anatomical T1-weighted image data sets (thickness, 1 mm; FOV, 250 mm; TR = 2050 ms; TE = 3.93 ms) for VBM analysis were acquired from each subject.

### Pre-processing and functional imaging data analysis

Pre-processing and data analysis were performed using statistical Parametric Mapping software (SPM8; Wellcome Department of Cognitive Neurology, London, UK) implemented in Matlab (Mathworks Inc., Natick, MA, USA). Prior to analysis, the BOLD images of gradient-echo images, taken using an echo planar sequence, and three obtained T1-weighted images were re-aligned and re-sliced to the mean image of the series. BOLD images were co-registered to the participant's mean T1-weighted image, normalized against a standardized Montreal Neurological Institute (MNI) stereotaxic space to give images with 2-× 2-× 2-mm voxels. The short-term memory phase and updating memory phase of the task were regarded as different conditions and were separately modeled in the analysis. Only brain activities from the updating memory phase and not those from the short-term memory phase were included in the following analysis. A design matrix was fitted for each participant with one regressor for the WM phases of each task speed (Fast, Medium, or Slow) for each memory load (0-, 2-, 3-, or 4-back) as well as for the short-term memory phases of each task speed for each memory load (2-, 3-, or 4-back) using a standard hemodynamic response function (HRF). Six parameters obtained by rigid body correction of head motion were then regressed out by adding these variances to the regressor. The design matrix weighted each raw image according to its overall variability to reduce the impact of movement artifacts [28]. The design matrix was then fitted to each participant's data. After estimation, beta images were smoothed (10 mm full-width half-maximum) and taken to the second level or subjected to random effect analysis. This smoothing procedure was employed because it is recommended that spatially unsmoothed raw data are used with this method, and smoothing the beta images of unsmoothed raw data results in more independent data points for estimating the variance of the images [28].

We removed low frequency fluctuations using a high-pass filter with a cut-off value of 300 s. This high-pass filter cut-off value was chosen in this study because there were many conditions and using a lower high-pass filter cut-off value would cut the frequency of the model for each condition. We confirmed that using this value would leave the frequency of each model substantially untouched. Individual-level statistical analyses were performed using a general linear model (GLM).

Analyses were performed for estimates associated with the 0- and 2-back conditions (*i.e.*, excluding the 3- and 4-back conditions). This was to exclude the inverted U-curve effects or saturation effects [29] of brain activity, which appear when subjects are approaching their WM capacity limits, and also to exclude the possibility that substantial differences in error rates between conditions affect brain activity. Inclusion of the 2-back Fast condition and exclusion of the 3- and 4-back Fast conditions are supported by the results of a previous study [29]. In this previous study, accuracy of the 2-back condition was 88% and that of the 3-back condition was 81%. In this previous study, no decline was observed in brain activity in the 2-back condition, whereas apparent declines in brain activity were observed in the 3-back condition in some regions. In this study, accuracy of the 2-back Fast condition was 91.5% and that of the 3-back Fast condition was 85% (see the Results section). Thus, while a decline in brain activity due to reduced accuracy was not expected for the 2-back Fast condition, it certainly cannot be ignored for the 3-back Fast condition in some regions. While it is true that a subtle decline was observed in the 2-back Fast condition, findings from the previous study [29] suggest that an accuracy of >90% cannot deduce a drop in or saturation of brain activity, whereas this is not the case in conditions with an accuracy of 80% or so.

We initially performed 22 separate single-participant analyses, in which linear contrasts were used to identify region-specific condition effects (one subject was excluded from the analysis due to excessive motion during the fMRI scan, as described above). Contrast maps from individuals were entered into the second level of analysis and statistical inferences for each contrast were derived using a one-sample *t*-test. Data were subjected to a random effect analysis which allowed inferences derived from the sample size to be generalized to the population. The significance of each activation was estimated using distributional approximations from the theory of Gaussian fields [30]. Areas of activation were identified as significant if they passed a threshold of *P*<0.05, corrected for multiple comparisons at voxel-level F.W.E at the whole brain level.

The effect of memory load in WM tasks was obtained by subtracting the total of the 0-back conditions from all of the 2-back conditions (2-back−0-back). To show the areas commonly activated by increased memory load regardless of task speed effects, we used inclusive masking techniques. First, we identified regions with increased activity as memory load increased in each task speed condition using the following contrasts: (2-back Fast−0-back Fast), (2-back Medium−0-back Medium), and (2-back Slow−0-back Slow) (*P*<0.05, uncorrected). Then, among regions commonly identified by these three contrasts (“inclusive masking”), the (2-back−0-back) contrast was tested. The main effect of memory load in the WM task can be derived without considering the activation profile of the 1-back task (in another words, by comparing WM conditions with non-WM conditions), because the (2-back−1-back) contrast showed the same activated areas as the (1-back−0-back) contrast [31].

The main effect of task speed was obtained by examining the {(2-back Fast−2-back Slow)+(0-back Fast−0-back Slow)} contrast after inclusive masking with the (2-back Fast−2-back Slow) contrast and the (0-back Fast−0-back Slow) contrast (*P*<0.05, uncorrected).

Most importantly, an interaction analysis (*i.e.*, the interaction between speed and the nature of the task) was used to reveal the effects of WM-specific speed on brain activity. The effect of the speed of WM-specific cognitive processes was obtained by subtracting the effect of the task speed of simple sensorimotor tasks from the effect of the task speed of WM tasks {(2-back Fast−2-back Slow)−(0-back Fast−0-back Slow)}, after inclusive masking by the contrast (2-back Fast−2-back Slow) (*P*<0.05, uncorrected). This inclusive masking procedure was performed to detect regions of activity that showed the interaction effect {(2-back Fast−2-back Slow)−(0-back Fast−0-back Slow)} described above and also the effect of the task speed of the WM task (2-back Fast−2-back Slow). This confirmed that the interaction effect cannot be explained by the effect of the slowness of the 0-back task (0-back Slow−0-back Fast) alone. By varying stimulus frequency, one is varying the activity per unit time in diverse cognitive processes such as sensory processing, linguistic processing, object recognition, and so on. By subtracting the effect of the task speed of simple sensorimotor tasks from the effect of the task speed of WM tasks, we can rule out the possibility that these differences in the amount of diverse cognitive processes affect the brain activity of interest.

Additionally, the effect of error processing was estimated by using parametric modulation analyses to assess the effect of error processing on brain activity and to confirm that the effect of error processing alone cannot explain the effect of WM-specific speed on brain activity. In this parametric modulation analysis, the accuracy of each condition was put into the parameter of each condition. As each session had only two blocks per condition, in this analysis rendering the difference in this parameter for a single session almost meaningless, we first regarded and treated data from three sessions as data from one session. We next investigated the total effect of accuracy (negative correlations between brain activity and the error rate) and the error rate (positive correlations between brain activity and the error rate) for all conditions (in other words, the sum of the estimates for parametrically modulated models for all conditions, except those in which there were no errors). A similar analysis was not performed for reaction time, as reaction time is meaningless in 2-back conditions used in this study (subjects know what to push before the presentation of the stimuli in 2-back conditions).

### Connectivity data

**Figure 3:**
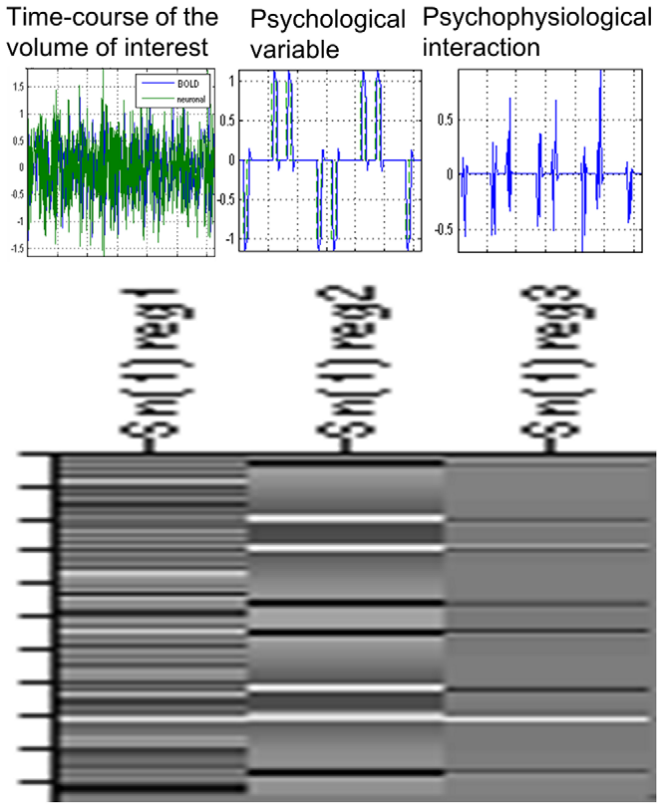
The schema of the design matrix used for PPI analysis of one session. The left column and left figure represent the time-course of the volume of interest (in case of analysis in this study, the right DLPFC). The middle column and middle figure represent the psychological variable (the effect of WM-specific speed; {(2-back Fast−2-back Slow)−(0-back Fast−0-back Slow)}. The right column and right figure represent PPI.

After having identified regions showing an effect of WM-specific speed on functional activity, we performed psychophysiological interaction (PPI) analysis [32] to identify the effect of this WM-specific speed on functional connectivity with regions of interest (ROIs). PPI analysis was performed as described elsewhere [33] using SPM8. The right dorsolateral prefrontal cortex (DLPFC) was chosen as ROI because it is a key node of the WM network and also because it is a part of ROI and the region where a significant result was observed in the analysis of functional activity, as described in the Introduction and Results sections. The coordinate of the peak voxel from the contrast for the effect of WM-specific speed {(2-back Fast−2-back Slow)−(0-back Fast−0-back Slow)} for the right DLPFC was used as a landmark for the individual seed voxel. A spherical ROI with a diameter of 6 mm was identified around the peak voxel (landmarks for the individual seed voxel described above) in the right DLPFC in the {(2-back Fast−2-back Slow)+(0-back Fast−0-back Slow)} contrast, which was derived from data that were not filtered to reduce the impact of movement artifacts [28]. The time series of each ROI was then extracted, and a PPI regressor was calculated as the element-by-element product of the mean corrected activity of this ROI and a vector coding for the differential task effect of WM speed-specific effects. In addition to the regressor that represents the interaction between the time series and the task, the main effects that contributed to that interaction (task and time series) were also included. Thus, the PPI regressor reflected the interaction between psychological variables {(2-back Fast condition−2-back Slow condition)−(0-back Fast condition−0-back Slow condition)} and the activation time-course of the right DLPFC. The schema and design matrix for this analysis are shown in Fig. 3. We also performed the same procedures for other psychological variables (2-back Fast−2-back Slow) to make mask images for use in the following procedures. The individual contrast images reflecting the effects of PPI on other brain areas were subsequently analyzed by one-sample *t*-tests. The threshold was set at *P*<0.05 (corrected for multiple comparisons at voxel-level F.W.E at the whole brain level) after inclusive masking by images reflecting a PPI of (2-back Fast−2-back Slow) (uncorrected *P*<0.05) of the ROIs (the right DLPFC). This inclusive masking procedure was performed to detect regions whose functional connectivity with the ROI showed not only the interaction effect {(2-back Fast−2-back Slow)−(0-back Fast−0-back Slow)} described above, but also the effect of the task speed of the WM task (2-back Fast−2-back Slow). It was also performed to confirm that the interaction effect could not be explained by the effect of the slowness of the 0-back task (0-back Slow−0-back Fast) alone.

### Volumetric data

Voxel-based morphometry (VBM) [23] was used to examine both the associations between individual differences in regional gray and white matter volume in brain regions and the ability to execute fast and more complex cognitive processes using WM. As described previously, three T1-weighted images were obtained from each subject and then re-aligned and re-sliced to the mean image of the series. This mean image was used for the VBM analysis to obtain an accurate morphological image. Data from one participant, previously excluded from the functional imaging analysis because of excessive motion, was included in the VBM analysis. Each subject's T1-weighted anatomical scan was segmented using the segmentation algorithm in VBM8 [34] with default parameter settings, but for two exceptions: affine regularization was performed in accordance with the averaged sized template and sampling distance (the approximate distance between sampled points when estimating the model parameters) was 1 mm. Segmented gray and white matter images underwent Jacobian modulation to adjust for the effects of spatial normalization. Images were smoothed with a 10-mm, full-width, half-maximum isotropic Gaussian kernel to the Gaussian random field model underpinning statistical inferences and an absolute threshold mask of 0.20 was applied to avoid partial volume effects. In whole-brain multiple regression analysis, we tested for associations between the accuracy of the 4-back Fast condition (the individual's ability to execute fast complex cognitive processes) and regional gray matter volume and white matter volume, after regressing out the reaction time for simple non-WM sensorimotor tasks (*i.e.*, 0-back Fast condition).

Data from the 2-back conditions and 0-back conditions, which were used for the fMRI analysis and the PPI analysis, were not used in the VBM analysis. This was to prevent differences in error rate affecting the results of the fMRI and PPI analysis conditions, among which accuracy rates were saturated (accuracy was close to the measurement ceiling) and differences were minimal. On the other hand, in VBM analyses, as long as we use multiple regression linear analysis, variables in the analysis should vary considerably. This is because if the accuracy rates of most of the subjects are saturated in the condition, they cannot be used as decent variables. In the 3-back Fast condition, ten out of 23 participants (and in the 2-back Fast condition, 15 out of 23 participants) showed accuracies that exceeded 90%, apparently showing saturation in performance of several subjects, while in the 4-back Fast condition, only one out of 23 participants showed accuracy that exceeded 90%. Thus the accuracy of the 4-back fast condition was used as a variable for the VBM analysis in this study.

The level of statistical significance was set at *P*<0.05, corrected for multiple comparisons at voxel-level FWE at the whole brain level. In this VBM analysis, ROI analysis was performed in addition to the abovementioned whole brain analysis. ROI was the right DLPFC because this is part of the total ROI and also the region where functional activity analysis revealed a significant result, as described in the Introduction and Results sections. In this ROI, the level of statistical significance was set at *P*<0.05 with a small volume correction for multiple comparisons (voxel-level FWE) in the mask image. The mask image was a 20-mm-radius sphere located around the peak voxel of the significant result in functional activation analysis of the effect of WM-specific speed. A relatively large sphere was used because of the possible subtle differences in the peaks of the two analyses that could have arisen from methodological differences between the two analyses. For example, the peak of functional activation might have been affected by the vessel, and the peak of morphological analysis might have been affected by regional morphology.

## Results

### Behavioral data in MRI

**Figure 4:**
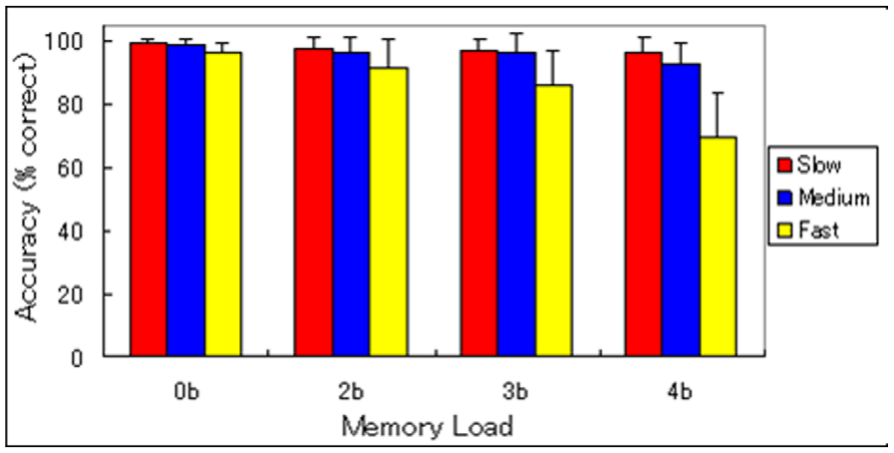
Behavioral results: accuracy across varying task speeds and memory loads. Error bars represent standard errors.

Questionnaires that were taken after the last scanning session confirmed that all the subjects could adhere to the instructed strategies (see Methods and Fig. 2). As shown in Fig. 4, a repeated measure ANOVA revealed that accuracy during the N-back task decreased significantly with increased memory load {F(3,66) = 43.76; *P*<0.001}. Participants made more errors overall at the fast speed (ISI = 1.2 s) compared to the medium or slow (ISI = 2.4 and 3.6 s, respectively) speeds {F (2, 44) = 64.31; *P*<0.001}. Also a significant interaction was observed between task speed and memory load effects {F (6,132) = 35.94; *P*<0.001}. At the slow speed, accuracy did not differ significantly between memory loads, whereas at the fast speed, accuracy decreased with increased memory load, and at the medium speed, accuracy of the 0-back load was significantly higher than that of the 4-back load. For the 0-back load, accuracy did not differ significantly between task speeds, whereas participants made more errors overall at the fast speed than at the slow speed for the 2-back load (however, for the difference in the accuracy of the WM specific speed contrast {(2-back Fast−2-back Slow)−(0-back Fast−0-back Slow 0), see below}. In the fast WM task, participants made more errors overall at the fast speeds than at the medium or slow speeds for the 3- and 4-back loads. Participants were less accurate in the fast WM tasks than in the slower WM tasks, which suggests that increased task speed in the WM task burdens the WM system. In the 3-back and 4-back fast tasks, accuracy fell below 90%, indicating that participants were facing their WM capacity limits. Thus, only estimates associated with the 0-back and 2-back conditions (*i.e.*, excluding the 3-back and 4-back conditions) were used in the analysis; this measure was taken to ensure that the inverted U-curve effects [29] in brain activity, which appear when participants are facing their WM capacity limits, did not affect our results and to exclude the possibility that substantial differences in error rates between conditions affect brain activity. Note that in the N-back task, without any updating operation, an accuracy rate of 50% is achievable, thus an accuracy rate that is considerably higher than chance performance (25%) does not necessarily mean subjects are doing the task properly. Among participants whose data were used for the fMRI analysis, no significant difference in the accuracy of the WM specific speed contrast {(2-back Fast−2-back Slow)−(0-back Fast−0-back Slow)} was found (*P*>0.05, one-sample t test). The mean of the difference in the accuracy of this contrast {(2-back Fast−2-back Slow)−(0-back Fast−0-back Slow)} was 2.6%, which amounted to less than one error per block of 28.8 seconds, indicating that the difference in the accuracy of this contrast was kept to a minimum. Furthermore, for effects of accuracy and error rate on functional activation, see the independent section below. The remaining conditions and the effects of WM capacity will be analyzed in a future report.

### Main effects of memory load and task speed on functional activation

**Figure 5:**
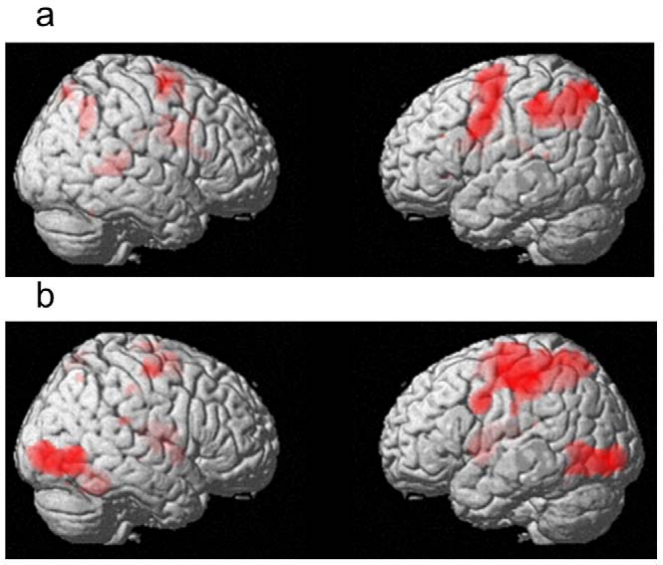
Main effects of task factors on functional activation. (a) Main effect of memory load in the WM task on functional activation. The results are shown with a threshold of *P*<0.005, uncorrected for visualization purposes. (b) Main effect of task speed regardless of task nature (non-WM task, WM task). The results are shown with a threshold of *P*<0.005, uncorrected for visualization purposes.

The main effect of memory load in the WM task (2-back *versus* 0-back) revealed a network including the bilateral DLPFC, left ventrolateral prefrontal cortex, supplementary motor area, left inferior and superior parietal lobule, and bilateral caudate (*P*<0.05, corrected for multiple comparisons at voxel-level F.W.E at the whole brain level; Fig. 5a). These areas are highly consistent with the areas identified in a previous meta-analysis of verbal N-back tasks [35]. These findings confirm the validity of our task for manipulating memory load in WM. The main effects of task speed {(2-back Fast−2-back Slow)+(0-back Fast−0-back Slow)} were revealed in regions including the bilateral occipital lobe, left pre- and post-central gyri, right fusiform gyrus, and right thalamus (*P*<0.05, corrected for multiple comparisons at voxel-level FWE at the whole brain level). However, when a more lenient threshold was applied, the main effect of task speed was revealed across a widespread network throughout the brain, including a broad range of frontoparietal regions that are critical for WM (*P*<0.005, uncorrected; Fig. 5b).

### Effect of WM-specific speed on functional activation

**Figure 6:**
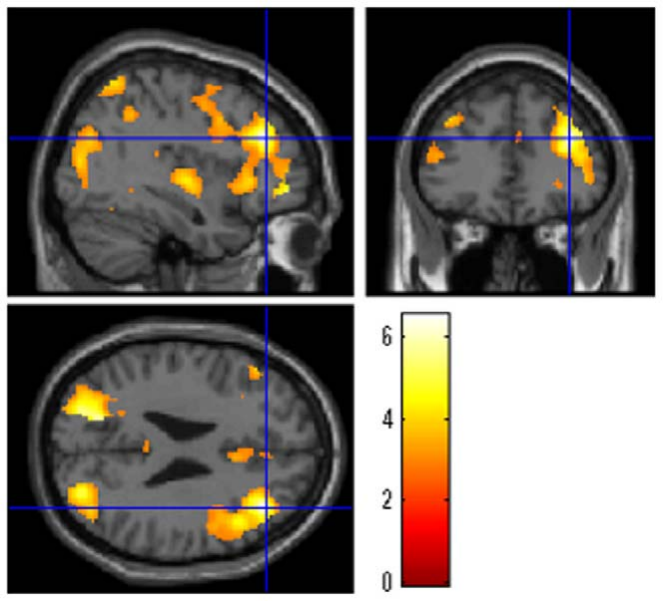
The effect of WM-specific speed on functional activation. A significant result was identified in the right DLPFC. The results are shown with a threshold of *P*<0.005, uncorrected for visualization purposes.

To identify the areas specifically activated by the increasing speed of WM-associated cognitive processes, an interaction contrast was derived by subtracting the speed effect of the 0-back task (a non-WM simple sensorimotor task) from that of the 2-back task (a WM task), yielding (2-back Fast−2-back Slow)−(0-back Fast−0-back Slow). Statistically significant activity was identified in the right DLPFC (*x, y, z* = 38, 36, 26, *t* = 6.53, *p* = 0.028 corrected for multiple comparisons at voxel-level F.W.E at the whole brain level; Fig. 6).

### Effects of accuracy and error rate on functional activation

The parametric modulation analysis revealed that there were no significant results relating to the total effect of accuracy or the error rate across all conditions. Next, we made an exclusive mask of the total effect of error rate of all conditions (uncorrected *P*<0.05) and confirmed that the result of the functional activation of the WM specific speed did not change with this exclusive mask.

### Functional connectivity analysis

Based on the activation elicited by WM-specific speed, we identified peak activation within the right DLPFC. PPI [32] was used to assess functional connectivity between the right DLPFC and other regions of the brain in relation to the effect of WM-specific speed.

**Figure 7:**
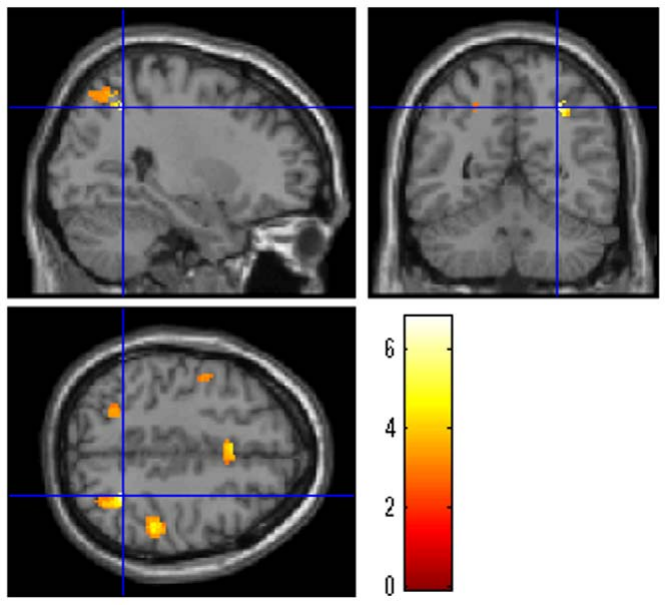
Increases in the functional interaction with the right DLPFC in response to the increases in the WM task speed. Increase in the WM task speed was specifically associated with the increased functional interaction between the right DLPFC (a key node of the WM network) and the right superior parietal lobe. The results are shown with a threshold of *P*<0.005, uncorrected for visualization purposes.

PPI analysis showed that the right DLPFC increased its functional connectivity with the right superior parietal lobe, specifically in response to the increases in the WM task speed (*x, y, z* = 28, −54, 46; *t* = 6.70; *P* = 0.038, corrected for multiple comparisons at voxel-level FWE at the whole brain level; Fig. 7). Therefore, the increased WM speed was specifically associated with the increased functional interaction between the right DLPFC and right superior parietal lobe, which are parts of the frontoparietal network.

### Performance of the fast WM task and gray and white matter volume

**Figure 8:**
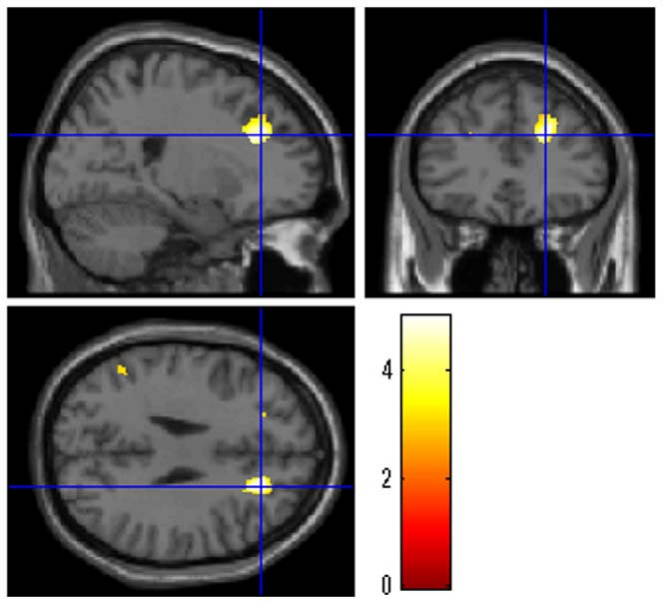
Gray matter correlates of the ability to execute fast cognitive processes in WM. Voxel-based morphometry was used to determine the relationship between regional gray matter volume and accuracy in the fast WM task, highlighting the effects in the right DLPFC. The results are shown with a threshold of *P*<0.005, uncorrected for visualization purposes. The peak voxel of this result corresponded well with the peak of WM-specific speed activation in the right DLPFC (distance, 13 mm). The subtle difference in the peaks of the two analyses might result from methodological differences in the two analyses. For example, the peak of functional activation might have been affected by the vessel, and the peak of morphological analysis might have been affected by regional morphology.

Next, we used VBM (see Methods) to examine the relationship between regional gray and white matter volume and performance on the fast WM task (*i.e.*, accuracy during the 4-back Fast condition). After controlling for the reaction time of a fast non-WM simple sensorimotor task (*i.e.*, the 0-back Fast condition), whole brain multiple regression analyses revealed that that participants' performance in the fast WM task was not significantly correlated with regional gray matter volume nor regional white matter volume in any of the regions. Next, we investigated whether regional gray matter volume and regional white matter volume in regions adjacent to the significant region that showed an effect of WM-specific speed were associated with the participants' performance in the fast WM task. Multiple regression analyses revealed that a WM region in the right DLPFC showed a significant correlation between the participants' performance in the fast WM task and regional gray matter volume (*x*, *y*, *z* = 23, 33, 28; *t* = 5.01; *P* = 0.012, corrected for multiple comparisons controlling for voxel-level FWE using a small volume correction within a 20-mm sphere around the peak of the significant result in functional activation; Fig. 8) but not between the participants' performance in the fast WM task and regional white matter volume.

## Discussion

The present study provided insight into the difference between the speed of simple sensorimotor cognitive processes and the speed of the more complex WM. Our initial hypothesis, that the involvement of the DLPFC and the network involving frontal and parietal regions is crucial when subjects engage in faster and more complex cognitive processes, was supported by our convergent approach. Our results showed that the increased speed of WM-specific cognitive processes was associated with the increased activation of the right DLPFC. We also showed that this increased speed of WM-specific cognitive processes was associated with heightened functional connectivity between the right DLPFC and right superior parietal lobe. Furthermore, GMV in the right DLPFC was correlated with participants' performance of fast WM tasks after correcting for the reaction times of the fast simple sensorimotor task. These are neural substrates that correspond to the differences between performing the simple tasks quickly and performing the complex tasks quickly. These differences probably explain why the speed with which subjects perform cognitively complex tasks is psychometrically different from the speed with which subjects perform simple tasks; and thus, why they associate more with psychometric intelligence.

One might think changing task speed changes subjects' response criterion from one that emphasizes accuracy to one that emphasizes speed, and these speed/accuracy criterion changes might affect neural activity or functional connectivity [36]. However, such changes are unlikely considering that accuracy merely changes among the conditions used in the functional activity or functional connectivity analysis and accuracy is well controlled near the upper limit (see Results). Thus, subjects increased their pace in the Fast conditions while they remained almost as accurate as they had been in the Slow conditions (for details of the subtle difference among conditions and its effect on functional activation, see Results).

### Effect of WM-specific speed on functional activation

Our hypothesis that the LPFC is crucial in the WM-specific speed effect was supported by our present result. Differences of accuracy between conditions (increased error rate in the fast WM condition) could not explain this result for two reasons; because the effect of error rate on brain activity did not affect the activity of the identified areas and because only low-load conditions, in which accuracy was almost saturated (accuracy was close to the measurement ceiling), were analyzed in this study and no significant differences in accuracy existed for the WM-specific speed contrast {(2-back Fast−2-back Slow)−(0-back Fast−0-back Slow)}.

Furthermore, children with slower processing speeds tend to augment the activation of the right middle frontal gyrus as the WM load increases [37]. In other words, the right middle frontal gyrus is progressively recruited as the WM load increases and places a greater burden on the individual's processing speed. This interaction between speed and memory load in a WM task is consistent with our results regarding the involvement of the right DLPFC. This finding of functional activation also supports the methods of the present study which investigated the neural correlates of the difference between working memory speed and simple sensorimotor speed by investigating through manipulating the task condition as the two methods led to the same finding in this region. Similarly, the neural correlates of working memory obtained by activation studies through manipulating task conditions (memory load)[35], as well as the ones obtained by correlation studies that analyzed the association between working memory capacity and brain imaging measures [38], have substantial overlaps in lateral frontal and parietal regions. Therefore, these two methods (correlation studies using individual difference and studies manipulating task conditions) are able to help each other.

In addition, the LPFC's activity may mediate the psychological association between complex cognitive speed and psychometric measures of intelligence. The common involvement of the LPFC's activity in the difference between speed of simple and complex cognitive processes and psychometric measures of intelligence [18] is comparable to psychological studies showing that a cognitively complex processing speed task is more strongly correlated with psychometric measures of intelligence than is a simple processing speed task [2].

### Increased functional connectivity in response to WM-specific speed

The increased functional interaction between the right DLPFC and right superior parietal lobe in response to WM-specific speed is consistent with previous neuroimaging findings regarding psychometric measures of intelligence and functional connectivity [19]. Higher functional connectivity between the DLPFC and other frontoparietal regions at rest has been associated with superior psychometric intelligence scores [19], suggesting that the increased functional interaction among frontoparietal regions is crucial for complex cognitive processes. Consequently, higher functional connectivity between the right DLPFC and right superior parietal lobe is a common neural correlate of the resting state of individuals with higher psychometric measures of intelligence and the difference between complex cognitive speed (WM speed) and simple sensorimotor speed. Furthermore, because a cognitively complex processing speed task is more strongly correlated with psychometric measures of intelligence than is a simple processing speed task [2], [3], [4], [5], our results may indicate that the common involvement of the increased functional connectivity between the right DLPFC and right superior parietal lobe mediates this relationship between psychometric measures of intelligence and the performance of psychological speed measures of various complexities.

### VBM

Our results regarding the significant positive correlation between GMV in the right DLPFC and performance on the fast WM task are comparable to those of a previous structural imaging study showing that psychometric measures of intelligence are linked with GMV in the LPFC in older adults [20]. Together with our morphological findings, individuals with increased GMV in the LPFC are characterized by both better performance of fast WM tasks and higher psychometric measures of intelligence. This notion is congruent with the psychological finding that complex cognitive speed is strongly correlated with psychometric measures of intelligence [2], [4], [5]. Furthermore, our results are consistent with a previous finding [39] that RT in a simple task was not correlated with the volume of GM, but that the faster RT of the memory task, which was presumably more cognitively complex, was positively correlated with more GM in the DLPFC in middle aged subjects. However, that study found a significant correlation in the left DLPFC, unlike our finding in the right DLPFC. The possible causes of this incongruence between studies include differences in subjects' ages and in the tasks.

We did not account for individual differences in the performance of slow WM tasks or executive function tasks in the VBM analysis. Therefore, we cannot rule out the possibility that the VBM analysis reflected individual differences in executive function. Nor can we determine whether the right DLPFC is important for the performance of slower WM capacity. Considering these limitations, future studies examining individual differences in the performance of slow WM tasks and executive function tasks and their correlations with GMV in the right DLPFC are required.

### Other limitations

Although, N-back tasks are typically used in fMRI studies, it is probably for the reason that they allow subjects to do tasks continuously with the same cognitive load though the properties of the tasks may differ from those of other WM tasks (such as continuous updating). However, N-back tasks can constantly tap the neural substrates of WM [17], [35], thus combining this preferable characteristic with the suitability of the N-back tasks in fMRI studies, we believe that N-back tasks are the most suitable tasks for the purpose of this study. An additional requirement of the present task is that subjects maintain the numbers to which the response buttons are assigned in WM, as was the case in most fMRI tasks. However, since we gave subjects an extensive practice period for the fMRI task, we believe the burden and the effect of this additional requirement was kept to a minimum. Finally, linear increase in speed might cause a non-linear increase in activity. In the present whole brain analyses, countless voxels were observed, but we could only investigate the effect of pre-specified contrasts or weighting of conditions (instead of fitting a curve to the given data). The present contrast and weighting might not tap the effect of task speed in the most efficient manner, at least in some regions. Although this is common to whole brain analyses involving weighting of conditions and data, it should be noted as a limitation.

### Summary

Traditionally, it is known that the speed with which subjects perform simple cognitive tasks and the speed with which subjects perform more complex cognitive tasks have different psychometric properties, such as strength of association with psychometric intelligence, vulnerability to aging, and vulnerability to the circadian rhythm. We have used functional and structural neuroimaging techniques to identify the brain regions responsible for the difference between the speed of cognitive processes in WM and the speed of simple sensorimotor processes. Our findings suggest that the involvement of the right DLPFC and the increased functional connectivity between the right DLPFC and right superior parietal lobe are critical for the speed of WM-specific cognitive processes, and that the morphological basis of DLPFC underlies the execution of fast cognitive processes in WM. These neural bases may underlie psychometric differences between the speed with which subjects perform simple cognitive tasks and the speed with which subjects perform more complex cognitive tasks, as well as explain the previous traditional psychological findings regarding this matter.
